# Key enablers and barriers to ICU nurses’ professional identity: a qualitative study

**DOI:** 10.3389/fmed.2025.1695617

**Published:** 2025-11-14

**Authors:** Tingting Tang, Jie Yang, Chunyan Wang, Yang Zou, Yixiu Liu

**Affiliations:** 1 Department of Critical Care Medicine, West China Hospital, Sichuan University, Chengdu, China; 2 West China School of Nursing, Sichuan University, Chengdu, China; 3 School of Business, Guilin University of Electronic Technology, Guilin, China

**Keywords:** intensive care unit, ICU nurses, ecological systems theory, professional identity, multidimensional framework, qualitative research

## Abstract

**Objectives:**

To explore the key enablers and barriers influencing the professional identity among Intensive Care Unit (ICU) nurses.

**Methods:**

This is a qualitative study that employed a purposive sampling approach and was conducted in the intensive care units (excluding pediatric ICUs) of two tertiary hospitals in Chengdu, China. One-on-one, semi-structured in-depth qualitative interviews were conducted with 37 ICU nurses. The Ecological Systems Theory (EST) provided the theoretical framework for this study. Thematic analysis approach was used to analyze the data.

**Results:**

We developed four themes to explain the qualitative data: Individual Motivation and Achievement System (IMAS), Team Collaboration and Support System (TCSS), Technology and Societal Support System (TSSS), Sociocultural and Values System (SVS). IMAS highlighted intrinsic motivation, professional competence, and patient satisfaction as key enablers, while lack of research and innovation capabilities, excessive workload, frequent night shifts, and misunderstanding from patients’ families constitute major challenges. TCSS reflected the importance of effective teamwork and multilevel support, with improper collaboration identified as a barrier. TSSS emphasizes the dual role of advanced technology and public health emergencies, representing both opportunities and challenges. SVS reflects the beneficial effect of positive social perceptions and cultural values, contrasted with social cognitive biases as obstacles.

**Conclusion:**

This study underscores enablers and barriers influencing ICU nurses’ professional identity within the EST framework. Professional competence and a sense of irreplaceability enhance intrinsic motivation, whereas excessive workload and promotion pressure undermine stability. Interprofessional collaboration and multilevel support reduce burnout, while technological advances both improve efficiency and increase skill-related anxiety. Interventions such as optimizing workload and work environment, providing professional development, improving performance evaluation, and implementing hospital policies and broader healthcare reforms in China may strengthen professional identity, improve job satisfaction, and support the sustainable development of ICU nursing practice.

## 1 Introduction

Professional identity refers to an individual’s recognition of their social role within their field of work. It encompasses professional values and role identity while reflecting the sense of recognition and belonging experienced by individuals at organizational and societal levels (1–3). In contemporary clinical practice, patients admitted to the Intensive Care Unit (ICU) typically presents with life-threatening injuries or critical illnesses, requiring skilled healthcare professionals equipped with specialized nursing competencies, comprehensive knowledge, empathetic care, advanced technological resources, and multidisciplinary support (4). Therefore, within this highly specialized, technology-intensive, high-pressure, and complex clinical environment, ICU nurses’ positive professional identity holds significant multidimensional value. It not only supports nurses in managing complex care tasks effectively but also enhances their self-confidence and professional self-esteem, improves nursing quality, job satisfaction, and team collaboration efficiency, and improves their retention in the profession (1, 4, 5).

Since the onset of COVID-19, the professional identity of ICU nurses has gradually received increasing attention (5–11). Existing studies have primarily addressed mental health (4), occupational stress (4, 12), moral distress (13, 14), and competency development (5), highlighting challenges and coping mechanisms in high-intensity work environments. While these studies provide valuable insights, several limitations remain. First, previous studies have predominantly focused on individual perceptions, values, or psychological aspects of nurses’ professional identity (6, 7, 14), reflecting a relatively narrow perspective and providing limited systematic investigation within a theoretical framework. Second, most existing research has relied on quantitative survey methods (6, 9), which are valuable for identifying general trends but provide limited insight into the contextual and in-depth aspects of nurses’ work experiences and professional identity. Third, cultural and contextual factors specific to Chinese ICU settings have been underexplored. Collectively, these limitations indicate the need for qualitative research that considers multidimensional influences and China’s cultural context to better understand ICU nurses’ professional identity.

In the Chinese context, ICU nurses face not only high-pressure clinical environments (14), but also social and cultural factors such as the public’s limited understanding of critical care nursing, low recognition of their professional status, and strained doctor-nurse relationships (15–17). These factors further exacerbate the conflict between “occupational value” and “professional identity,” impacting nurses’ mental health and career development.

To deeply explore the mechanisms and influencing factors behind the formation of ICU nurses’ professional identity, this study introduces Ecological Systems Theory (EST) as its theoretical framework. EST, proposed by U. S. developmental psychologist Bronfenbrenner (18), emphasizes that individual development is situated within a multi-level, dynamically interacting ecological environment. This environment includes four levels: microsystem (i.e., individual, family, work environment), mesosystem (interactions among microsystems), exosystem (indirect effects of environmental changes), and macrosystem (broader sociocultural expectations like cultural values, social norms). This study focuses on ICU nurses, positing that their professional identity is not formed in isolation, but as a result of long-term interactions between the individual and their surrounding environment. In this ecological system, ICU nurses’ professional identity is shaped by various internal and external factors, including personal qualities, self-efficacy, emotional regulation, and other individual-level intrinsic factors, as well as interpersonal interactions, organizational culture, institutional norms, and societal recognition.

The Ecological Systems Theory (EST) is widely used in public health and education (19–21). EST, proposed by U. S. developmental psychologist Bronfenbrenner (18), emphasizes that individual development is situated within a multi-level, dynamically interacting ecological environment. This environment includes four levels: microsystem (i.e., individual, family, work environment), mesosystem (interactions among microsystems), exosystem (indirect effects of environmental changes), and macrosystem (broader sociocultural expectations like cultural values, social norms). EST emphasizes that individual outcomes cannot be understood in isolation, but rather emerge from the dynamic interactions among these systems. In the context of ICU nursing, professional identity is shaped by various internal and external factors, influenced not only by individual motivations and competencies but also by interactions with colleagues, organizational structures, and the broader cultural and social environment. EST provides a novel perspective to analyze the impact of the multilevel factors on the ICU nurses’ professional identity, enabling this study to systematically explore the formation and development of ICU nurses’ professional identity across individual, organizational, and sociocultural contexts.

Guided by the EST, this study aims to conceptualize ICU nurses’ professional identity as the outcome of interactions across multiple environmental levels, including the individual, family, work environment, organizational, and cultural systems. Through semi-structured interviews and framework-oriented coding analysis, the study seeks to identify factors influencing the formation and development of nurses’ professional identity, while providing both theoretical foundations and practical insights for enhancing professional identity and supporting career development.

## 2 Materials and methods

### 2.1 Design

This study employed a qualitative approach, guided by EST, to explore the enablers and barriers to ICU nurses’ professional identity. This approach emphasizes participants’ subjective experiences within specific sociocultural contexts and generate meaning through a collaborative dialog between researchers and participants. One-on-one, semi-structured in-depth interviews were used to collect qualitative data (22). Furthermore, the Consolidated Criteria for Reporting Qualitative Research (COREQ) checklist was used to guide the manuscript preparation (23).

### 2.2 Theoretical framework

**Figure 1:**
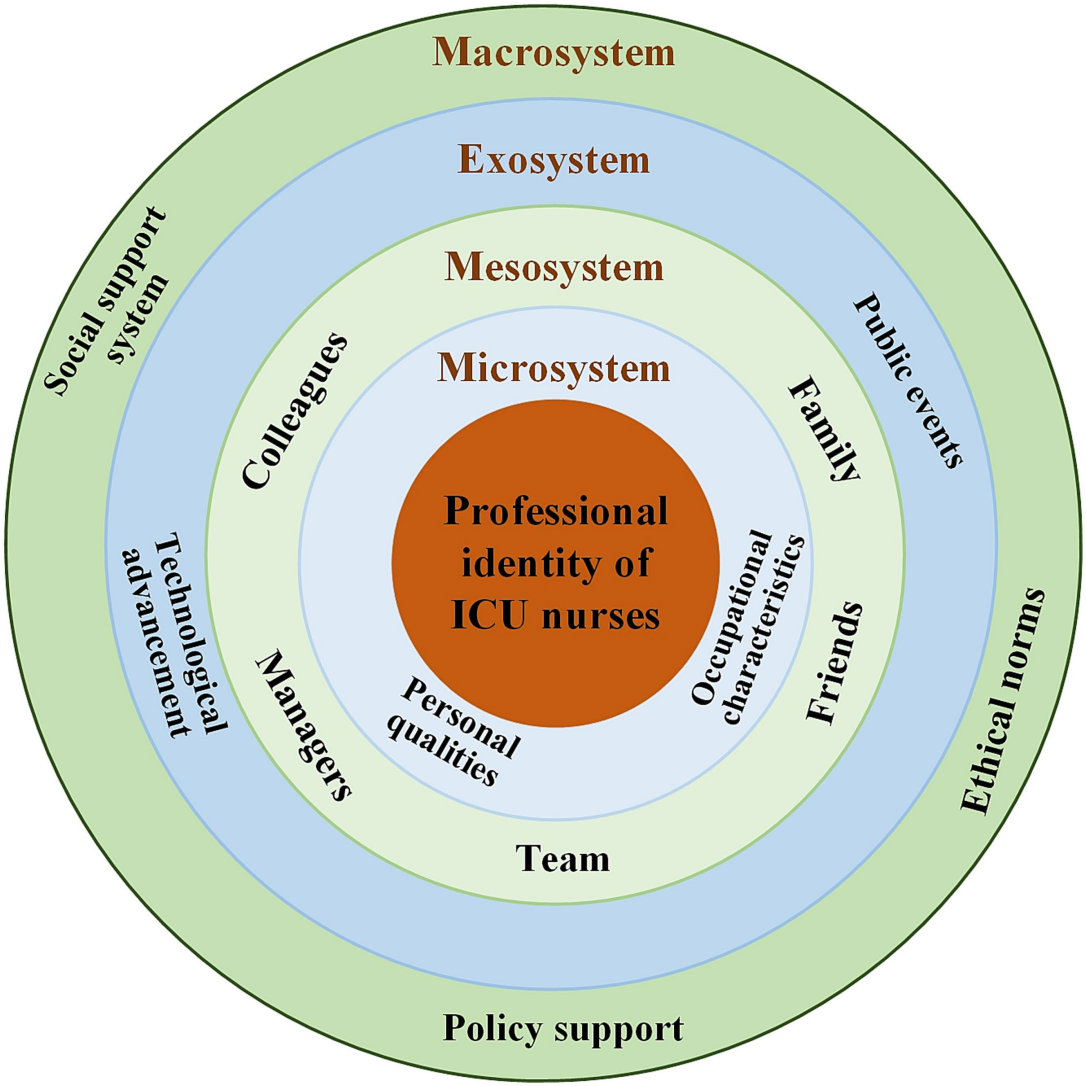
EST model.

The EST provided the theoretical framework for this study (18). According to the framework, the microsystem refers to the immediate environment that directly affects the individual, such as the nurse’s qualities, professional factors, and direct interactions with patients. The mesosystem is a system of interactions between multiple microsystems, such as the relationships between family members, friends, colleagues, leaders, and team collaboration. The exosystem does not directly affect the nurses but influences them through indirect mechanisms, such as technological advancements and public health events. The macrosystem encompasses broader socio-cultural, policy, and value factors, such as social support systems, professional ethics, and national-level institutional support. Figure 1 illustrates the framework of factors influencing ICU nurses’ professional identity constructed based on the EST model. It integrates four dimensions: microsystem, mesosystem, exosystem, and macrosystem, and clearly depicts the logical relationships among these dimensions.

### 2.3 Setting

This study was conducted in the intensive care units (excluding pediatric ICUs) of two tertiary hospitals in Chengdu, China. One of the public hospitals comprises eight critical care wards, a respiratory support and treatment center, and over 20 subspecialized teams dedicated to intensive monitoring, treatment, and nursing care. The ICU has a capacity of over 200 beds and employs more than 500 medical and nursing staff, and admits over 10,000 critically ill patients annually. The other private hospital’s ICU is a specialized unit dedicated to the centralized management and critical care of patients with severe infections. It has a capacity of 25 beds, 2 medical teams and over 70 medical and nursing staff. The interviews were conducted between March and April 2025.

### 2.4 Participants and recruitment

A purposive sampling strategy was adopted (24), and participants were recruited from the ICUs of one public tertiary hospital and one private tertiary in Chengdu, China. Inclusion criteria required participants to be registered nurses currently working in adult ICUs with at least 1 year of ICU clinical experience. Exclusion criteria included nurses on leave, those rotating from non-ICU departments, nursing interns, residents in training, and visiting nurses. To ensure diversity across key characteristics, participants were purposively selected to vary in ICU work experience, professional title, position, and frequency of night shifts, aiming to obtain information-rich cases relevant to the study objectives, with a total of 37 participants were included. Participation was voluntary and written informed consent was obtained. All participants in this study were informed that participation was entirely voluntary and that declining or withdrawing would not affect their employment or professional evaluation. Recruitment and informed consent procedures were conducted solely by the research team to minimize the influence of hierarchical power dynamics.

### 2.5 Researcher characteristics and reflexivity

The interviewer and analysis team comprised five researchers: T. Tang and Y. Zou, both registered nurses with master’s degrees and training in qualitative research; J. Yang, Ph. D., professor and doctoral supervisor with extensive experience in qualitative research methodologies; C. Wang, with relevant clinical and research experience; and Y. Liu, a master’s student with training in qualitative research. Their clinical expertise and research skills enabled a deep understanding of ICU nurses’ perspectives, enhancing the quality and depth of the interviews. Specifically, before the interviews, the primary interviewers (Y. Liu) reflected on their roles to remain neutral and avoid imposing their views. During the interviews, they clarified participants’ understanding and feelings to ensure accurate interpretation. In data analysis, T. Tang and Y. Zou reviewed and compared text segments to ensure consistency and coherence in their interpretations. To ensure credibility, the coders had to set aside their personal experiences and perceptions and fully immerse themselves in the data. All themes and categories were derived from the data. J. Yang provided guidance on maintaining research rigor throughout the interviews and analysis, while C. Wang contributed to coding, data organization, and verification, supporting transparency and reliability of the analytic process.

### 2.6 Data collection method

The interviews were conducted in private offices or teaching rooms. The settings were flexibly selected based on participants’ psychological comfort, aim to create a familiar, quiet, and private environment conducive to emotional expression. The interview guide was developed based on the EST (18), relevant literature (10, 25–33), and expert consultations. It consisted of eight open-ended questions and one optional supplementary question (see Supplementary material). To ensure its appropriateness in addressing the study objectives (34), a pilot test was conducted with two participants, and no revisions were deemed necessary. The research team members with advanced academic backgrounds in medicine and nursing, who have received systematic theoretical training and practical experience in qualitative research methods, with no direct reporting relationships between the interviewers and the participants. Each interview was audio-recorded on-site with the participant’s prior written consent. A professional voice recorder served as the primary recording device, with a mobile phone app used as a backup to prevent data loss. During the interviews, researchers took field notes in real time, documenting key phrases, vocabulary, and nonverbal cues such as facial expressions, gestures, tone, emotional responses. During the subsequent analysis, the researchers integrated field notes to capture and reconstruct key verbal and non-verbal information from the interviews. Throughout the data collection process, the research team continuously assessed data saturation. The absence of new themes and data saturation guided the determination of the sample size (35). After analyzing the data of the 34th participant, no additional new information emerged, and three more participants were invited to ensure data saturation. No participants refused to participate or withdrew halfway. Within 24 h after each interview, the recordings were verbatim transcribed, and the transcripts were double-checked by two researchers to ensure accuracy.

### 2.7 Data analysis method

The data were analyzed using a thematic analysis approach, following Braun and Clarke’s six-phase framework (36). This involved (1) familiarizing ourselves with the data, (2) generating initial codes based on predefined theoretical constructs, (3) searching for themes, (4) reviewing themes, (5) defining and naming themes, and (6) producing the final report. Audio recorded data were transcribed into verbatim, following the principle of context preservation (37) to accurately capture participants’ intended meanings, including emotional expressions, pauses, and tone. The transcripts were coded using NVivo 20. These transcripts were read repeatedly to gain a comprehensive understanding of each participant’s narrative. To ensure accuracy and contextual integrity, two researchers (Y. Zou and T. Tang) conducted preliminary coding, developed an initial coding manual, and independently reviewed each transcript multiple times, and consensus was reached on the divergent parts through discussion and guided review by qualitative research experts (J. Yang) to ensure the stability of the coding results. Significant statements related to ICU nurses’ professional identity were extracted, and meanings were formulated from these statements. These meanings were then organized into categories and further grouped into subthemes corresponding to the four dimensions of EST. The themes were integrated into a comprehensive description of the factors influencing professional identity, identifying the core structures that underpin nurses’ professional behavior. Throughout the analytical process, the researchers maintained reflexivity by continually examining their own assumptions, emotional respo nses, and potential biases to ensure analytical rigor and minimize interpretive distortion (37).

### 2.8 Rigor

To ensure the transparency and trustworthiness of the analytical process, an audit trail was established, including raw recordings, verbatim transcripts, coding notes, and theme development documentation. An audit trail was maintained throughout the research process, including raw data, coding records, analytic memos, and documentation of methodological decisions. All transcripts and field notes were anonymized and stored in a password-protected database accessible only to the research team. All procedures were independently conducted and cross-checked by two researchers. The extracted themes and categories were returned to 37 participants for confirmation, and all agreed with the interpretation.

### 2.9 Ethics statement

The study was conducted in accordance with the ethical standards of the 1964 Declaration of Helsinki and received approval from the Biomedical Ethics Committee of West China Hospital, Sichuan University (Ethics Approval No. 2025453). All participants were made aware that the participation was voluntary. Written consent was obtained, and anonymity was maintained. To protect participants’ privacy, pseudonyms in the format of P01–P37 (P for Participant) were used during data analysis.

## 3 Results

### 3.1 Participant characteristics

Thirty-seven participants were included in the final analysis, comprising 30 females (81%). The mean age of participants was 32.76 years (SD = 5.54), ranging from 25 to 45 years. Their work experience in ICU ranged from 1 to 21 years, with a mean of 9.24 years (SD = 5.58). Thirteen participants (35%) held intermediate professional titles, 57% hold junior titles despite one-third accruing ≥10 years’ ICU experience, and only 8% have progressed to senior/managerial rank, revealing a compressed career ladder. Three-quarters earn ≤15,000 RMB monthly, while married nurses predominate (76%) yet single staff shoulder the heaviest night-shift load (8 ~ 12/month). Educational attainment is high (92% bachelor’s, 8% master’s), but advanced degrees remain under-leveraged in junior roles. The interviews lasted approximately 30–40 min. The general Information of the participants are detailed in Table 1.

**Table 1:** General information table of research subjects (*n* = 37).

Serial number	Gender	Age	ICU work experience (years)	Professional title	Marital status	Monthly income (RMB)	Educational level	Job position	Number of night shifts per month
P1	Female	26	5	Junior	Unmarried	10,000–15,000	Bachelor	Primary nurse	10
P2	Female	30	6	Junior	Married	5,000–10,000	Bachelor	Primary nurse	6–8
P3	Female	33	7	Intermediate	Married	5,000–10,000	Bachelor	Primary nurse	12
P4	Female	36	14	Junior	Married	10,000–15,000	Bachelor	Team leader	10–11
P5	Male	33	11	Junior	Married	5,000–10,000	Master	Primary nurse	4
P6	Female	34	13	Junior	Married	10,000–15,000	Bachelor	Primary nurse	10–11
P7	Female	29	10	Junior	Married	10,000–15,000	Bachelor	Primary nurse	8–10
P8	Female	35	12	Intermediate	Married	7,000	Bachelor	Primary nurse	6–10
P9	Female	28	5	Junior	Married	5,000–10,000	Bachelor	Primary nurse	8–10
P10	Male	31	10	Junior	Unmarried	5,000–10,000	Bachelor	Nursing team leader	8–10
P11	Female	42	2	Senior	Married	15,000–20,000	Bachelor	Nurse manager	2–4
P12	Female	32	10	Intermediate	Married	8,000	Bachelor	Primary nurse	2–4
P13	Female	41	21	Junior	Married	10,000–15,000	Bachelor	Team leader	2–4
P14	Male	28	5	Junior	Married	1,000–15,000	Bachelor	Primary nurse	4–10
P15	Female	45	15	Intermediate	Married	15,000–20,000	Bachelor	Team leader	0–4
P16	Male	26	3	Junior	Unmarried	8,000–10,000	Bachelor	Primary nurse	10
P17	Female	27	4	Junior	Married	5,000–10,000	Bachelor	Primary nurse	10
P18	Female	41	17	Intermediate	Married	5,000–10,000	Bachelor	Team leader	0
P19	Male	25	3	Junior	Unmarried	10,000	Bachelor	Primary nurse	8–10
P20	Female	31	8	Intermediate	Married	6,000	Bachelor	Primary nurse	8–9
P21	Female	41	19	Intermediate	Married	15,000	Bachelor	Team leader	8–10
P22	Male	30	6	Junior	Married	10,000–13,000	Bachelor	Primary nurse	10
P23	Female	25	2	Junior	Unmarried	8,000–10,000	Bachelor	Primary nurse	10
P24	Female	36	1	Intermediate	Married	15,000–20,000	Bachelor	Primary nurse	8–10
P25	Female	41	17	Intermediate	Married	15,000–20,000	Bachelor	Primary nurse	0–4
P26	Female	27	1	Junior	Unmarried	10,000	Master	Primary nurse	4–8
P27	Male	34	11	Intermediate	Married	13,000–15,000	Bachelor	Team leader	8–10
P28	Female	39	17	Intermediate	Married	15,000–20,000	Bachelor	Team leader	8–10
P29	Female	37	16	Intermediate	Married	15,000–20,000	Bachelor	Team leader	8–10
P30	Female	27	6	Junior	Unmarried	8,000–10,000	Bachelor	Primary nurse	8–10
P31	Female	27	6	Junior	Unmarried	8,000–10,000	Bachelor	Primary nurse	8–10
P32	Female	35	16	Junior	Married	10,000–15,000	Bachelor	Primary nurse	6
P33	Female	37	11	Intermediate	Married	5,000–10,000	Bachelor	Primary nurse	4–5
P34	Female	34	11	Junior	Married	5,000–10,000	Bachelor	Primary nurse	10
P35	Female	29	8	Junior	Married	5,000–10,000	Bachelor	Team leader	9–11
P36	Female	34	12	Junior	Married	10,000–15,000	Bachelor	Team leader	8–11
P37	Female	26	1	Junior	Unmarried	5,000–10,000	Bachelor	Primary nurse	10–12

“Junior” refers to Junior professional title, including registered nurse and senior nurse; “Intermediate” refers to Intermediate professional title, including supervisory nurse; “Senior” refers to Senior professional title, including associate chief nurse and chief nurse.

### 3.2 Themes and subthemes

**Figure 2:**
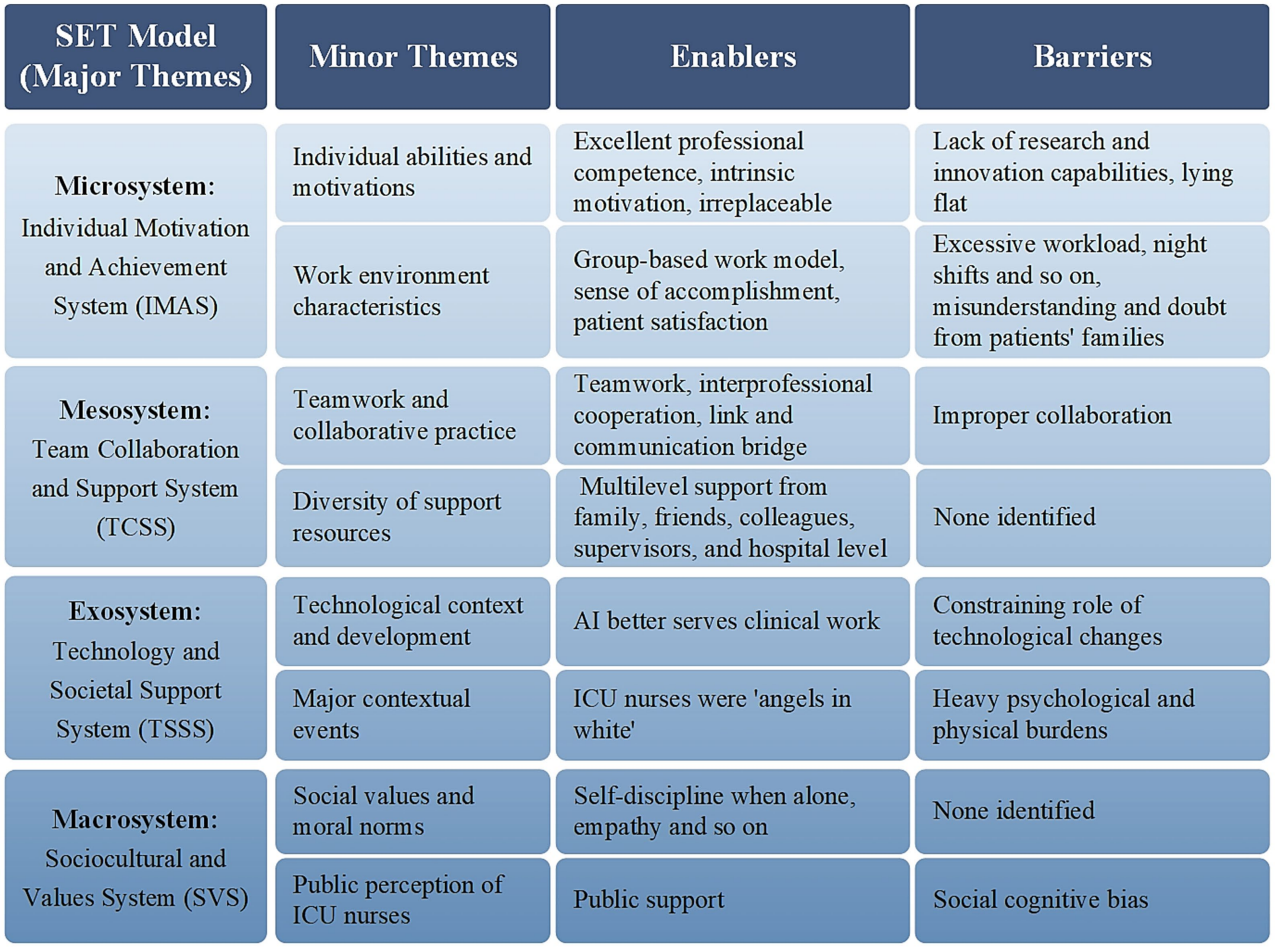
Major and minor themes.

Four major themes were identified based on the EST model, including the Individual Motivation and Achievement System (IMAS), Team Collaboration and Support System (TCSS), Technology and Societal Support System (TSSS) and Sociocultural and Values System (SVS). Figure 2 integrates the four major themes and their eight minor themes, and illustrates the enablers and barriers corresponding to each minor theme.

#### 3.2.1 Theme 1: individual motivation and achievement system

##### 3.2.1.1 Sub theme 1: individual abilities and motivations

The critical condition and rapid changes in ICU patients, often accompanied by emergencies, require timely and effective treatment. Therefore, ICU nurses must possess excellent emergency rescue skills and a comprehensive, in-depth knowledge base to make quick, accurate judgments and decisions in complex and critical situations. Compared to nurses in general wards, ICU nurses are held to higher standards in terms of professional skills, emergency response, and comprehensive handling capabilities. Participants reported a strong sense of professional knowledge and practical skills than nurses in general wards, described by one participant as:

“*Compared to general wards, for example, we draw arterial blood gasses ourselves. In general wards, when they cannot draw blood gasses, they call us… Also, we are the ones who perform CRRT in the ICU (expressing great pride)*.” (P2)

These professional knowledge and skills enable them to perform nursing duties in general departments with ease and approach critical patients with greater confidence. One participant stated:

“*I believe that as an ICU nurse, I have a broader and deeper mastery of professional knowledge and skills compared to nurses in general departments. During my rotations in other departments, this knowledge and skillset has been immensely helpful, allowing me to handle emergency care for critically ill patients in general wards*.” (P5)

Participants in this study consistently emphasized that sufficient intrinsic motivation was a key driving force enabling ICU nurses to maintain progress and development under high-pressure conditions. Intrinsic motivation refers to the internal drive that originates from an individual’s own needs, interests, and values, rather than external rewards or incentives (38). It plays a vital role in sustaining enthusiasm and perseverance in the face of challenges, fostering continuous growth and self-improvement. One nurse suggested:

“*ICU work is all about urgency and critical illness. If we only perform basic tasks like administering injections and IVs without observation or integrating learning, we end up being passive… So, we need to build our professional skills to proactively make predictive and evaluative judgments*.” (P14)

With the rapid advancement of science and technology, the application of artificial intelligence (AI) in healthcare has continued to expand, raising widespread concerns about whether critical care nursing roles may eventually be replaced. In this study, participants generally believed the unique clinical reasoning and humanistic care provided by ICU nurses are irreplaceable, and that AI cannot fully substitute for these essential aspects of nursing. One participant commented:

“*AI may gradually take over routine or standardized operational tasks, but it cannot replace the warmth and empathy that are unique to nurses, those human-centered roles that require a personal touch*.” (P25)

The demanding nature of ICU nurses’ clinical workload often leaves them with limited time and energy for research and innovation, thereby hindering their advancement in professional titles. Many nurses attributed this situation to their own perceived lack of competence and gradually adopted a passive or disengaged attitude, often described colloquially as “lying flat,” with one nurse stating:

“*Regarding title promotion, the requirements are quite high nowadays, and they often involve research-related work. For clinical nurses like us, research is definitely a weak area. Many people feel that since they cannot achieve it, they simply give up and ‘lying flat’. If I do not pursue it, I will not get it, and that means I do not have to try. I’ll just focus on doing my daily work well*.” (P3)

##### 3.2.1.2 Sub theme 2: work environment characteristics

Medicine is a profession dedicated to saving lives and alleviating suffering, bearing a noble and honorable mission. As guardians of life, healthcare professionals are expected to possess a strong sense of mission and responsibility. In this study, participants expressed a general appreciation for the ICU work model. One participant noted:

“*I actually think working in the ICU is quite good. The team has strong cohesion, and I really like the group-based work model*.” (P24)

Whenever patients’ conditions improve, participants reported experiencing a deep sense of professional achievement and mission, which further strengthened their professional identity. One participant remarked:

“*When patients are successfully transferred out of the ICU, both doctors and nurses here feel a sense of accomplishment, it feels like we carry a halo. There’s a strong sense of mission*.” (P4)

Patient satisfaction has long been a central focus of hospital quality improvement. From the perspective of nurses, patient satisfaction represents the highest form of affirmation for their hard work and serves as a powerful source of motivation to continue delivering high-quality care. In this study, one participant highlighted that patient satisfaction was an important source of motivation:

“*Most of the patients transferred out of our ICU express a high level of satisfaction, both the patients and their families. Our unit has been actively promoting early rehabilitation, offering individualized programs such as karaoke and mahjong depending on the patient’s condition… Many patients feel that their time in the SICU was actually enjoyable, not as distressing as they imagined. Some patients have even said they miss their time in the ICU after discharge, because they did not have to worry about sudden changes in their condition, they just needed to maintain a good mood and get enough rest. That’s been very encouraging for me*.”

(P30)

The core obstacles to ICU nurses’ professional identity may stem from the inherent constraints of the profession itself, which are closely tied to the nature and characteristics of critical care work. During the interviews, participants identified multiple factors that hinder the development of their professional identity, including excessive workload, frequent night shifts, low compensation, intense performance evaluations, pressure related to professional title and academic advancement, and a growing number of training sessions and administrative tasks. These longstanding and multifaceted challenges significantly undermine nurses’ motivation and hinder their professional growth.

One nurse mentioned that frequent night shifts and heavy workload brought significant stress:

“*The workload is heavy, and night shifts are frequent. Sometimes it’s extremely exhausting and physically stressful.*” (P1)

One nurse remarked that the intensive workload in the ICU is disproportionate to the level of remuneration:

“*ICU has the highest turnover rate because of the enormous pressure. You feel like you are giving everything, but the reward is not proportional. We do the hardest work but receive the lowest pay. Sometimes when our unit is understaffed, we all have to work under high intensity, yet the compensation remains unfair….*”

One participant frankly expressed that she do not enjoy working in the ICU, but continue to do so out of life necessities:

“*To be honest, I do not really enjoy working in the ICU. The work is stressful, and although the pressure itself might seem minor, the compensation does not match my efforts. Considering that I am married and have children, if I were to resign, I would still need to find another job, so I feel compelled to continue, making compromises along the way. If it becomes unbearable, then I would resign, this is essentially my mindset…*.” (P2)

One participant reported experiencing pressure related to various assessments, examinations, and professional title promotions:

“*In medicine, we often say ‘lifelong learning’, but it feels like we are constantly being examined, tested, and assessed… There’s also pressure from title promotion, here we are expected to balance clinical work, teaching, and research. If you do not meet all the criteria, promotion is impossible.*” (P11)

Another participant mentioned being burdened by various training sessions and additional tasks:

“*If it’s just work-related fatigue, I can tolerate it. But when we are constantly assigned extra tasks and various training sessions without additional time or resources, it becomes overwhelming. I just wish they would stop piling on more duties.*” (P20)

The ICU operates under a closed management model, which leads to patients and their families lacking a comprehensive and in-depth understanding of the ICU’s work patterns and content. One participant mentioned that they often face misunderstandings and doubts from patients’ families:

“*We often hear family members say: ‘You are ICU nurses, providing critical care, but it seems you are not as good or as meticulous as ordinary wards or family caregivers.’ They think patient care is just basic daily care, but that’s not the case. Besides fundamental and daily nursing, we focus more on ICU-specific specialized care. So family members’ understanding is often partial or even lacking. They frequently wonder why patients’ hands and feet are tied up, or why patients do not respond when spoken to. There is still a lot of misunderstanding.*” (P11)

#### 3.2.2 Theme 2: team collaboration and support system

##### 3.2.2.1 Sub theme 1: teamwork and collaborative practice

ICU work relies heavily on team collaboration, and the degree of cooperation directly affects the quality and efficiency of care. Within the healthcare team, ICU staff are often regarded as exemplars of unity and collaboration. One participant emphasized the importance of teamwork:

“*The team is very important. Whatever you do, whether you succeed, fail, or even make mistakes, the team silently supports you. There is a saying: ‘If the people around you are full of positive energy, you are very likely to become a positive person as well’. So, the team is crucial. Regardless of success or failure, having a team that supports you makes you feel very warm.*” (P32)

In collaboration with clinical physicians, respiratory therapists (RTs), rehabilitation technicians, and other professionals, ICU nurses play an essential role in patient care and team coordination due to their professional competence and unique advantages. This not only improves the overall efficiency of medical work but also provides strong support for the sustainable development of the ICU, underscoring their irreplaceable value. One participant regarded ICU nurses as playing an important role in interprofessional teamwork:

“*I think the ICU is a team that highly emphasizes multidisciplinary collaboration. Whether it is doctors, nurses, therapists, or RTs, none can be missing. Only through cooperation can the work be done well. Each role has its division of labor, and nurses are an indispensable part. Only when every part is done well can the whole job be done well.*” (P26)

One participant viewed ICU nurses as the link and communication bridge:

“*Medical care is essentially a team effort. In our daily work, we need to communicate more with the medical team. In interprofessional collaboration, ICU nurses can serve as a bridge, such as acting as a communication link between patients and doctors, thereby better serving the patients.*” (P17)

However, one participant believed that ICU nurses hold the lowest status among doctors, nurses, and technicians:

“*…and another thing is that, actually, it shows that we (ICU nurses) are at the lowest level among the three groups, doctors, nurses, and technicians (bitter laugh).*” *(P16)*

ICU work requires strong team collaboration and a high degree of tacit understanding and coordination among healthcare staff. However, improper collaboration is difficult to completely avoid in practice. When teamwork is hindered, not only does work efficiency suffer, but nurses’ professional identity is also significantly undermined. One nurse noted as:

“*Sometimes in clinical work, some senior staff feel that ‘this is not my business’ or ‘this is not my responsibility’. Their first reaction is not to solve the problem but to excuse themselves. Often, you want to tell them how to handle the problem next time or remind them what to do next, but this kind of mindset cannot reach consensus. The message you want to convey and what they receive differ greatly.*” (P14)

##### 3.2.2.2 Sub theme 2: diversity of support resources

During the interviews, some participants mentioned that they received multifaceted support from the hospital, leadership, colleagues, family, and friends, which served as important sources of support and strength in their work.

One nurse mentioned support from the hospital, leadership, and colleagues:

“*Support from the hospital, colleagues, or leaders helps me overcome difficulties at work…*” (P7)

Two participants remarked support from family and friends:

“*Support from my parents and family helped me get through difficult times and allowed me to fully dedicate myself to work.*” (P7)

“*When I encounter difficulties, I talk to a close friend who might give me some helpful advice, helping me get out of the predicament or at least alleviating my tension and anxiety at that moment.*” (P21)

#### 3.2.3 Theme 3: technology and societal support system

##### 3.2.3.1 Sub theme 1: technological context and development

The development of advanced technologies, represented by AI, has injected new vitality into the field of critical care nursing. AI not only plays an important role in improving nursing quality and work efficiency but also promotes the intelligentization and precision of nursing practice. Moreover, the widespread application of AI in ICU clinical practice further strengthens ICU nurses’ professional identity and drives the deepening development and continuous advancement of critical care nursing. One participant expressed that AI helps “liberate” nursing manpower, reduce workload, and enhance efficiency:

“*Advanced medical technology can drive the development of the entire nursing industry. I think AI can reduce a lot of workload, and replace some of our inefficient, mechanical, and repetitive tasks, assist us in making better decisions… and eliminate some highly burdensome tasks, making (clinical) work more refined.*” (P31)

Technological advancement is often a double-edged sword, while it improves work efficiency, it may also bring a series of challenges. For example, although the application of AI in critical care nursing reduces the workload of ICU nurses, it can also lead to nurses developing dependency and complacency, which in turn may cause deterioration in their operational skills, increase their sense of professional insecurity, and potentially hinder their individual development. One nurse said:

“*…I feel this might weaken my professional identity because it seems like robots do everything now, and there’s hardly anything left for me to do. With AI applications becoming so widespread, I feel like I have no space to apply my skills. It makes me feel more insecure, like one day I might be unemployed.*” (P9)

One participant shared a unique perspective:

“*Sometimes I feel that overly advanced technologies are used merely to prolong life, but they do not necessarily (ensure the quality of life). I sometimes discuss this issue with doctors in our unit. In some cases, when (technology) becomes too advanced, it may not always be a good thing…perhaps things should be allowed to take their natural course.*” (P20)

##### 3.2.3.2 Sub theme 2: major contextual events

During the COVID-19 pandemic, numerous moving and exemplary deeds emerged, embodying the spirit of “disasters are ruthless, but humanity is compassionate.” ICU nurses played a critical role, shouldering most of the care for critically ill patients and becoming the main force on the frontline. While bearing this honor, they also faced tremendous occupational risks. During this challenging period, their courage and dedication further enhanced their professional identity as ICU nurses. One participant mentioned:

“*Nurses used to lack social recognition…people thought nurses only gave injections and infusions. But since the pandemic, perceptions have changed, especially toward ICU nurses. Now, everyone considers ICU nurses as ‘angels in white’ who save lives alongside doctors, gaining recognition from society and family, which also gives me a sense of achievement.*” (P17)

The COVID-19 was an unprecedented battle filled with uncertainty, confusion, and fear. During this special period, ICU healthcare workers remained steadfast at their posts amid anxiety and apprehension, facing unprecedented challenges. Under immense pressure from the pandemic, they not only encountered intense workloads but also bore heavy psychological and physical burdens. These factors became significant obstacles to ICU nurses’ development of professional identity. One nurse noted:

“*I remember when the pandemic fully opened up, our unit originally had 35 beds, but we kept adding more, we received nearly 40 to 50 patients… and their conditions were increasingly severe, one worse than another. So during that period, the nursing pressure, both psychological and physical, was definitely many times higher than usual… I felt really exhausted during that time.*” (P33)

#### 3.2.4 Theme 4: sociocultural and values system

##### 3.2.4.1 Sub theme 1: social values and moral norms

Professional values and ethical standards are critical to the quality and safety of nursing care and represent essential principles that every healthcare institution and the entire healthcare sector must rigorously uphold. In this study, starting from the perspective of professional values and ethical standards, participants emphasized that ICU nurses need to possess multifaceted professional qualities. Among these, one participant highlighted the spirit of integrity in solitude “self-discipline when alone” and patient-centeredness:

“*I think it’s not just ICU nurses, ordinary nurses also need to emphasize the spirit of integrity in solitude. It is an essential quality for all nurses. Of course, being patient-centered is the foundation for all nurses as well.*” (P5)

Additionally, One nurse mentioned the necessity for ICU nurses to have empathy and selfless dedication:

“*I believe one must have integrity and a spirit of dedication. For example, when caring for patients, you need to have compassion, right? And if you also have empathy, you can view things from the patient’s perspective… In short, your professional ethics determine your boundaries.*” (P7)

Moreover, some participants noted that maintaining a rigorous work attitude, respecting life, protecting patient privacy, and showing care and responsibility toward patients were also important expressions of individual professional quality:

“*Working in the ICU demands rigor, love, and strong responsibility.*” (P17)

“*…it means being more careful and prudent in your work, always maintaining respect for life.*” (P22)

“*… protect their (refers to patients) privacy while also firmly holding one’s convictions, working diligently and responsibly to improve professional quality.*” (P23)

“*In ICU, you must have a high sense of responsibility. Much of what we do must be worthy of our conscience. Moreover, my own moral motivation drives me to do many things beneficial to patients, even beyond medical orders or assigned tasks.*” (P31)

##### 3.2.4.2 Sub theme 2: public perception of ICU nurses

The external support system is the foundation for ICU nurses’ career establishment and success. A sound support system not only helps nurses better cope with high-pressure environments but also enhances their professional confidence, identity, and sense of achievement. One participant noted:

“*If they (refer to public) fully support or understand my work, I will be doubly responsible. This makes me feel that my work is very meaningful and important.*” (P26)

However, some participants believed that certain people in society look down on ICU nurses:

“*…I think there are a small number of people in society who look down on ICU nurses…*.” (P17)

“*…I feel they (refers to general public) still holds some prejudice against ICU nurses. They think the ICU is a place for hard labor, exhausting, busy, demanding, and poorly paid…*.” (P15)

Although ICU nurses play a critical role in critical care, public understanding of their work content and professional value remains relatively limited. Some participants thought that society often misunderstands the role of ICU nurses, neglecting their professional competence and responsibilities in clinical judgment, critical patient management, and interdisciplinary collaboration. This external cognitive bias makes them feel underestimated. As one nurse noted:

“*I think many people see the ICU as a very tiring place, but they do not understand the level of technology now. They only know ICU nurses wash patients’ faces, clean them, and turn them over, but they do not realize that every step involves expertise. For example, when we touch patients’ skin, we assess their volume status; when washing faces, we observe pupils, mouths, noses, these are all observational processes that people do not understand and often misunderstand.*” (P15)

One participant noted that the low social status of nursing in China discourages some nurses from working in the field domestically:

“*Compared with nursing professions abroad, the social status of nursing in China is still relatively low, which causes many capable nurses around me to choose to work overseas where the pressure is less, making me less willing to pursue this career.*” (P19)

## 4 Discussion

This study offers a novel perspective on the factors influencing ICU nurses’ professional identity. While themes such as workload, leadership, and social recognition have been discussed in previous research, situating these findings within the institutional and cultural context of Chinese ICUs reveals deeper underlying mechanisms. For example, the tiered hospital system in tertiary hospitals, medical insurance payment reforms, performance evaluation in public hospitals, and the “14th Five-Year Plan” healthcare policies not only shape nurses’ career choices and professional identity but also influence team collaboration and motivational dynamics (39, 40). In addition, developments in artificial intelligence and the impact of the COVID-19 pandemic have introduced new challenges to ICU nurses’ professional identity, providing a novel analytical perspective. Theoretically, grounded in the EST framework, this study systematically integrates multiple dimensions, overcoming the limitations of previous single-perspective studies and highlighting the complexity and context-specific nature of ICU nurses’ professional identity.

Within the IMAS framework, excellent professional competence, intrinsic motivation, the irreplaceability of the position, patient satisfaction, and sense of professional accomplishment collectively constitute core supports for professional identity. Among these factors, “excellent professional competence” functions as the driving force underpinning the others. Previous research has demonstrated a strong correlation between nurses’ skill development and a more positive professional outlook (41, 42). It is indisputable that possessing rich professional knowledge and skills is a fundamental standard for any profession (5). Nursing is a highly technical profession demanding rigorous theoretical knowledge and practical skills (43). Olausson et al. (44) noted that patient-centered nursing techniques and equipment often become “extensions of the nurse’s body, outstretched arms, and eyes,” forming a safety guarantee for both patients and nurses. Meanwhile, Cederwall et al. (45) and Crocker and Scholes (46), in their studies on patient weaning from mechanical ventilation, explained how nurses utilize their advanced clinical skills and knowledge to provide patient-centered care that facilitates successful weaning. Thus, ICU nurses demonstrate a significant competitive advantage in the nursing profession through their outstanding professional competence. This capability not only strengthens their perception of the irreplaceability of their roles but also serves as a vital source of intrinsic motivation, thereby robustly supporting their sustained investment and positive performance in enhancing patient satisfaction and achieving professional accomplishment. This aligns with the findings of Slettmyr et al. (47), who suggested that the sense of achievement and engagement derived from professional identity improves nurses’ job satisfaction, benefiting their overall well-being and the provision of high-quality patient care. Notably, while “position irreplaceability” reinforces nurses’ professional value and role identity, it may paradoxically act as a latent trigger for occupational burnout under conditions of inadequate human resources. This highlights the need for managers to carefully balance workload demands with the provision of professional value recognition.

Conversely, Excessive workload, night shifts and so on, lack of research and innovation capabilities, and misunderstand and doubt from patients’ families constitute significant barriers. Similar to Mousazadeh et al. (5), who identified unprofessional behavior as an impediment to professional identity formation, the long-term neglect of nursing’s professional status and public distrust in nursing knowledge have consistently constituted major barriers to building a positive professional identity. It is well known that ICU work environments are high-pressure and fast-paced, with nurses frequently facing heavy workloads, bed shortages, and staffing constraints (14, 48), all of which intensify physical and psychological stress. The demands of managing critically ill patients and responding to emergencies require rapid nurse responsiveness, raising the bar for emergency response capabilities and team collaboration (12). Moreover, the unique physical limitations of ICUs in China (e.g., confined spaces, equipment overcrowding) and systemic organizational and environmental challenges must not be overlooked (4). Some participants mentioned the significant pressure from excessive workload, frequent night shifts, evaluations, various trainings and additional non-clinical tasks, as well as the stress associated with promotion and academic advancement in this study. The cumulative long-term effect of these factors has further destabilized ICU nurses’ professional identity. This phenomenon is also closely associated with the broader social context in China, characterized by a large population base contributing to a high total number of critical illness, an accelerating pace of societal operations, and ongoing reforms across various sectors. Such an environment places increasing demands on the nursing profession for greater specialization and diversification.

Within the TCSS framework, team collaboration and diverse support systems identified play a significant role in shaping the professional identity of ICU nurses. Efficient interdisciplinary teamwork not only enhances nurses’ satisfaction but also provides a supportive context for the development and consolidation of their professional identy. Active communication between physicians and nurses, collaborative decision-making, and effective interaction within multidisciplinary teams are regarded as key mechanisms for improving the quality of care (4, 45). Cederwall et al. (45) highlighted the pivotal role of team collaboration in the process of ventilator weaning. Moreover, high levels of participatory practice have been identified as effective strategies for promoting team cooperation and nurse empowerment. Such empowerment encourages nurses to actively shape their work environment, thereby reinforcing their professional roles and contributing to a stronger sense of identity within interprofessional teams (49). Fostering active interaction among nurses, as well as between nurses and professionals in other roles and functions, facilitates the exchange of knowledge, skills, and experiences. This, in turn, promotes the development of a more cohesive and influential professional nursing community (50). Conversely, barriers to collaboration represent significant negative factors. Poor communication, unclear role boundaries can lead to role conflict, thereby weakening nurses’ sense of professional identity and fostering ambiguity or fragmentation in how they perceive their roles. Participants consistently emphasized in this study, multilevel support, from family, peers, leadership, and organizational systems, was described as essential in maintaining their commitment to their roles. In daily clinical practice, the emotional support and encouragement provided by colleagues and supervisors were described as instrumental in enabling nurses to take on challenging responsibilities. Moreover, positive peer relationships were reported to improve job satisfaction and reduce turnover rates (4).

Within the TSSS framework, both technological progress and public health crises exhibit a “double-edged sword” effect. On the one hand, technological empowerment improves clinical efficiency and accuracy, enhancing nurses’ sense of professional fulfillment. For example, AI, by offering real-time data analytics and early warning scoring, significantly boosts decision-making capacity and can handle vast amounts of data more efficiently than humans (51, 52). This markedly improves the timeliness and effectiveness of clinical responses, providing robust support for the treatment of critically ill patients. On the other hand, technological advancements inadvertently raise professional standards and expectations, leaving some nurses feeling unable to keep pace with the rapid evolution of their field, thereby triggering anxiety about their own competencies. Recent studies have highlighted concerns over the potential erosion of clinical skills due to excessive reliance on AI technologies (51). Frequent dependence on large language models (LLMs) for executing critical tasks may impair the diagnostic and reasoning abilities of healthcare providers, particularly undermining the training and development of future professionals. The literature also documents increasing apprehension around the “deskilling” of medical personnel (53). These findings suggest that while embracing the convenience and efficiency brought by emerging technologies, careful consideration must be given to their rational use and inherent limitations. Maintaining a critical perspective on the potential long-term impact of technology is essential to achieving a sustainable balance between clinical efficiency and individual professional growth.

Additionally, large-scale global public health events, such as the COVID-19 pandemic, in the short term, have substantially heightened societal awareness of the nursing profession, serving as an external driving force for ICU nurses’ professional identity development. Research shows that nurses’ professional identity was generally strengthened during the pandemic (54, 55), largely due to increased public attention, heightened social recognition of nursing work, elevated perceptions of professional value, and mutual support within interdisciplinary medical teams (9, 56). However, these developments were accompanied by considerable challenges, including surging workloads, heightened risks of occupational trauma, and growing ambiguity surrounding professional roles. During the pandemic, ICU nurses were commonly exposed to fears of infection, insufficient protective resources, psychological stress, and burnout. These factors posed significant barriers to their job satisfaction and, to a certain extent, compromised the long-term stability of their professional identity (57).

Within the SVS framework, positive professional values and ethical principles offer a firm ideological foundation for the development of nurses’ professional identity. This aligns with the findings of Jakimowicz et al. (4), which suggest that affirmative nursing values play a critical role in supporting nurses, enhancing their job satisfaction, compassion, and professional growth. Meanwhile, adequate social support functions as a crucial external guarantee for the establishment of nurses’ professional identity. Such support not only alleviates the psychological pressures faced by nurses, but also enhances their sense of occupational belonging and societal value, thereby indirectly elevating their level of professional identity (58–60).

Conversely, social cognitive bias constitute a significant barrier. Although the nursing workforce consistently demonstrates strong professional loyalty and altruism in practice, ICU nurses remain subject to persistent public stereotypes about their professional roles (10, 61). In some studies, nurses have been portrayed primarily as individuals responsible for handling body fluids, administering injections, and providing basic patient care (62–64). As echoed in this study, one participant remarked: “*Many people think ICU nurses just administer IVs and clean up after patients.*” Such perceptions overlook the high technical, decision-making, and responsibility-oriented nature of ICU nursing, resulting in a systematic undervaluation of their role (11). This suggests that the public continues to lack an adequate understanding of the breadth and depth of the nursing profession, which contributes to widespread misconceptions about nurses’ roles and responsibilities (15, 16, 65). Another participant noted: “*The social status of nurses in China is still relatively low*,” a sentiment supported by findings from Chen et al. (17). This misalignment between societal expectations and professional realities not only undermines nurses’ sense of pride but also renders their identity more susceptible to external influences.

### 4.1 Strengths and limitations

This study provides several novel perspectives and insights into ICU nurses’ professional identity. First, through in-depth qualitative interviews, the study situates the findings within the institutional and cultural context unique to Chinese ICUs (e.g., the tiered hospital system in tertiary hospitals, medical insurance reforms, performance evaluation in public hospitals, and broader healthcare policy initiatives) as well as the country’s specific conditions (e.g., large population base, high number of critically ill patients), revealing multidimensional factors influencing nurses’ professional identity. Second, the use of the EST framework allows systematic integration of these multidimensional factors, facilitating a comprehensive understanding of the dynamics and complexity of nurses’ professional identity.

Nevertheless, several limitations should be acknowledged. First, participants were primarily recruited from a single city and specific hospital types, and findings may be influenced by local and institutional contexts, limiting generalizability to other regions or cultural settings. Second, data were primarily derived from self-reported narratives and interpretive analysis by the researchers. This introduces the potential for interview and interpretive bias, making it difficult to completely eliminate the influence of researcher subjectivity on the analytical process. Third, while the EST framework helped to systematically categorize the data, there is a potential risk of imposing theoretical structure, which may constrain data-driven theme generation. Finally, professional identity is a complex and evolving construct, and this study did not incorporate quantitative instruments or larger samples to further validate findings, leaving room for future theoretical expansion and empirical investigation.

## 5 Conclusion

This study, grounded in the EST, provides valuable insights into the factors influencing ICU nurses’ professional identity. Enablers factors (such as professional competence, intrinsic motivation, effective teamwork, multilevel support, advanced technology, and positive social perceptions) and barriers (including excessive workload, frequent night shifts, non-clinical duties, improper collaboration, academic and promotion pressures, technological and environmental challenges, and social cognitive bias) play a key role in shaping nurses’ professional identity. Key enablers include promoting skill development, enhancing team cohesion, ensuring organizational support, and increasing social recognition. Organizational and societal factors are also significant, indicating that interventions should consider hospital policies, broader healthcare reforms in China. Barriers can be mitigated through workload optimization, provision of professional development opportunities, and public advocacy initiatives (i.e., enhancing public awareness and recognition of ICUs and ICU nurses through science popularization educational). Identifying these factors can help nursing leaders and policymakers strengthen professional identity, improve job satisfaction, and ultimately enhance the quality of patient care. Future research should explore strategies to integrate these findings into workforce management and health policy to sustain professional growth and optimize ICU nursing practice.

## Data Availability

The original contributions presented in the study are included in the article/Supplementary material, further inquiries can be directed to the corresponding author.
