# Supplementary material for: Key enablers and barriers to ICU nurses’ professional identity: a qualitative study
Source: Front Med (Lausanne). 2025 Nov 14;12:1695617. doi: 10.3389/fmed.2025.1695617 (PMC12660275; doi:10.3389/fmed.2025.1695617)
Supplement: Supplementary file 1 [file Supplementary_file_1.docx]

**Supplementary file**

Consolidated criteria for reporting qualitative studies (COREQ): 32-item checklist

| **No** | **Item** | **Guide questions/description** | **Page no in manuscript/comment** |
| --- | --- | --- | --- |
| **Domain 1: Research team and reflexivity** |  |  |  |
| Personal Characteristics |  |  |  |
| 1. | Interviewer/facilitator | Which author/s conducted the interview or focus group? | P7 (4.6 Interviews) |
| 2. | Credentials | What were the researcher's credentials? *E.g. PhD, MD* | P9 (4.8 Rigour and Reflexivity) |
| 3. | Occupation | What was their occupation at the time of the study? | P9 (4.8 Rigour and Reflexivity) |
| 4. | Gender | Was the researcher male or female? | P9 (4.8 Rigour and Reflexivity) |
| 5. | Experience and training | What experience or training did the researcher have? | P9 (Rigour and reflexivity) |
| Relationship with participants |  |  |  |
| 6. | Relationship established | Was a relationship established prior to study commencement? | P7 (4.6 Interviews) |
| 7. | Participant knowledge of the interviewer | What did the participants know about the researcher? e*.g. personal goals, reasons for doing the research* | The participants did not know any of the researchers. However, all participants knew that the interview was for research purposes. |
| 8. | Interviewer characteristics | What characteristics were reported about the interviewer/facilitator? e.g. *Bias, assumptions, reasons and interests in the research topic* | None.Participants were introduced to the research using the ethics approved Explanatory Statement and Consent Form. |
| **Domain 2: study design** |  |  |  |
| Theoretical framework |  |  |  |
| 9. | Methodological orientation and Theory | This study was grounded in the Social Ecological Theory (SET) model and adopted an exploratory descriptive qualitative approach. Data were collected from the participants’ perspectives to obtain in-depth insights into the relevant themes, without being constrained by predetermined assumptions. The aim of such research is to explore and describe participants’ experiences and perceptions. Thematic analysis was employed to analyze the data. | P5(4.1 Design)  P6(4.2 Theoretical framework) |
| Participant selection |  |  |  |
| 10. | Sampling | How were participants selected? *e.g. purposive, convenience, consecutive, snowball* | P7 (4.4 Participants and recruitment) |
| 11. | Method of approach | How were participants approached? e*.g. face-to-face, telephone, mail, email* | P7 (4.6 Interviews) |
| 12. | Sample size | How many participants were in the study? | P1 (Results)  P10 (5.1 Particpant characteristics) |
| 13. | Non-participation | How many people refused to participate or dropped out? Reasons? | None of the participants withdrew during the interview process |
| Setting |  |  |  |
| 14. | Setting of data collection | Where was the data collected? e*.g. home, clinic, workplace* | P7 (4.6 Interviews) |
| 15. | Presence of non-participants | Was anyone else present besides the participants and researchers? | Nil, not relevant. |
| 16. | Description of sample | What are the important characteristics of the sample? *e.g. demographic data, date* | P10 (5.1 Particpant characteristics)  Described in Tables 1 (General Information Table of Research Subjects)P11 |
| Data collection |  |  |  |
| 17. | Interview guide | Were questions, prompts, guides provided by the authors? Was it pilot tested? | P7 (4.6 Interviews) |
| 18. | Repeat interviews | Were repeat interviews carried out? If yes, how many? | No repeat interviews were carried out |
| 19. | Audio/visual recording | Did the research use audio or visual recording to collect the data? | P7 (4.6 Interviews) |
| 20. | Field notes | Were field notes made during and/or after the interview or focus group? | P7 (4.6 Interviews) |
| 21. | Duration | What was the duration of the interviews? | P10 (5.1 Particpant characteristics) |
| 22. | Data saturation | Was data saturation discussed? | P7 (4.6 Interviews)  Throughout the data collection process, the research team continuously assessed data saturation. Recruitment was discontinued when no new significant themes emerged and narrative patterns became repetitive in subsequent interviews |
| 23. | Transcripts returned | Were transcripts returned to participants for comment and/or correction? | No member checks were carried out as this was not relevant to the study aims or rationale for the study. Nor was it practical. |
| **Domain 3: analysis and findings** |  |  |  |
| Data analysis |  |  |  |
| 24. | Number of data coders | How many data coders coded the data? | P8 (4.7 Data analysis) and P9 (4.8 Rigour and reflexivity) |
| 25. | Description of the coding tree | Did authors provide a description of the coding tree? | Not applicable. |
| 26. | Derivation of themes | Were themes identified in advance or derived from the data? | Themes and sub-themes were derived based on the SET model and through both inductive and deductive approaches to data analysis. |
| 27. | Software | What software, if applicable, was used to manage the data? | NVivo software was used P8 (4.7 Data analysis) |
| 28. | Participant checking | Did participants provide feedback on the findings? | Not applicable. |
| Reporting |  |  |  |
| 29. | Quotations presented | Were participant quotations presented to illustrate the themes / findings? Was each quotation identified? e*.g. participant number* | Quotations provided under P10 (5 Findings) section |
| 30. | Data and findings consistent | Was there consistency between the data presented and the findings? | Yes, quotes were provided verbatim. Quotations provided under Findings section |
| 31. | Clarity of major themes | Were major themes clearly presented in the findings? | Yes, P12 (5.2 Themes and subthemes) |
| 32. | Clarity of minor themes | Is there a description of diverse cases or discussion of minor themes? | Yes, P12 (5.2 Themes and subthemes) |
