# Supplementary material for: Key enablers and barriers to ICU nurses’ professional identity: a qualitative study
Source: Front Med (Lausanne). 2025 Nov 14;12:1695617. doi: 10.3389/fmed.2025.1695617 (PMC12660275; doi:10.3389/fmed.2025.1695617)
Supplement: Supplementary file 2 [file Supplementary_file_2.docx]

Interview guide

| Main questions |
| --- |
| - What factors in your work environment have contributed to the development of your professional identity? |
| - What factors have hindered the development of your professional identity? |
| - How do you perceive your professional knowledge and skills as an ICU nurse, and how does this perception influence your professional identity? |
| - What do you think is the role of ICU nurses within interprofessional teams, and how does this role positioning influence your professional identity? |
| - How have advanced medical technologies in the ICU influenced your understanding of your professional role? |
| - Have you been involved in the care of COVID-19 patients? If so, how did your experiences during the pandemic affect your perception of ICU nursing as a profession? |
| - In your work, how have your personal values and professional ethics influenced your sense of professional identity? |
| - How do you think societal perceptions of ICU nurses affect your development of professional identity? |
| - Is there anything else you would like to add regarding your professional identity? |
